# Bilateral pontine brachium lesions in one autoantibodies directed against MOG positive patient: A case report

**DOI:** 10.1097/MD.0000000000039278

**Published:** 2024-08-09

**Authors:** Fan-Ya Sun, Jia Shen, Le Ding, Bo Zhu, Naibing Gu, Zhiqin Liu, Zhengli Di, Xiao-Tao Jia

**Affiliations:** a Department of Neurology, The Affiliated Xi’an Central Hospital of Xi’an Jiaotong University College of Medicine, Xi’an, Shaanxi, People’s Republic of China.

**Keywords:** MRI, myelin oligodendrocyte glycoprotein (MOG), pontine brachium, treatment

## Abstract

**Rationale:**

Myelin oligodendrocyte glycoprotein (MOG) antibody-related disease is a relatively recent entity in inflammatory demyelinating disease. Its clinical presentation varies in severity and the lack of specific imaging features makes it easy to misdiagnose. We now report the case of a MOG antibody-positive patient who presented with diplopia and dizziness, and whose brain magnetic resonance imaging (MRI) showed abnormal signals in the bilateral pontine brachium.

**Patient concerns:**

A previously healthy 52-year-old woman presented with diplopia and dizziness, and was hospitalized 4 days after onset.

**Diagnoses:**

Brain MRI demonstrated abnormal hyperintense signals in the bilateral pontine brachium on T2-weighted fluid attenuated inversion recovery imaging. MRI enhancement showed abnormal enhancement foci in bilateral pontine brachium and pons. Cerebrospinal fluid examination showed Oligoclonal IgG bands were negative. The IgG index was normal, and serum aquaporin-4 antibody was negative, while serum MOG-Ab was positive (1:100). In conjunction with a positive serum MOG antibody and exclusion of other diseases, diagnosis of MOG antibody-related disease was made.

**Interventions:**

Intravenous methylprednisolone followed by oral corticosteroids.

**Outcomes:**

Symptoms resolved completely. At 4-month follow-up. Follow-up after 4 months showed disappearance of the abnormal signal in the left pontine brachium and diminution of abnormal high signal in the right compared to the previous one, and there was no recurrence 1 year after the onset of the disease.

**Lessons:**

If brain MRI indicating bilateral, multiple, and diffuse abnormal signals in the pontine brachium, and a discrepancy between the clinical symptoms and the imaging severity, a diagnosis of demyelinating disease should be considered highly probable. In such cases, anti-MOG antibody testing is essential for further defining the etiology. The clinical phenotype and imaging manifestations of MOG antibody-positive brainstem encephalitis may lack sufficient specificity to be readily identifiable. Timely diagnosis and early glucocorticoid therapy are beneficial in improving prognosis and preventing recurrence.

## 1. Introduction

Myelin oligodendrocyte glycoprotein (MOG) antibody-related disease (MOGAD) is a relatively new entity in the spectrum of inflammatory demyelinating disease. Common presentations of MOGAD include optic neuritis, myelitis, meningoencephalitis, and brainstem encephalitis, and it overlaps with multiple sclerosis (MS) and neuromyelitis optic spectrum disorders (NMOSD). Imaging in patients with MOGAD is frequently characterized by multifocal, bilateral involvement with indistinct lesion demarcation, particularly in T2-weighted fluid attenuated inversion recovery. However, previous studies have shown that MOGAD is devoid of distinctive imaging characteristics. Here, we report a patient with MOG antibody positivity who initially presented with diplopia and dizziness, exhibited abnormal signals in the bilateral pontine brachium on magnetic resonance imaging (MRI). This finding contributes to the understanding of the imaging characteristics of MOGAD and suggests that MOGAD should be considered as a potential diagnosis when similar imaging features are observed.

## 2. Case report

**Figure 1.**
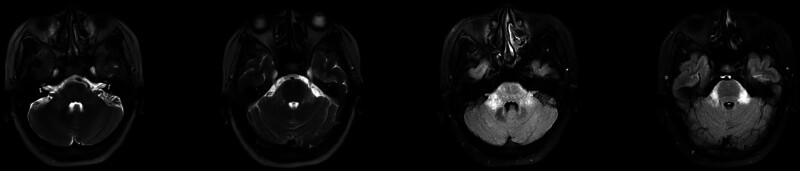
The brain MRI showed abnormal hyperintense signals with diffused borders in the bilateral pontine brachium, predominantly on the right side.

**Figure 2.**
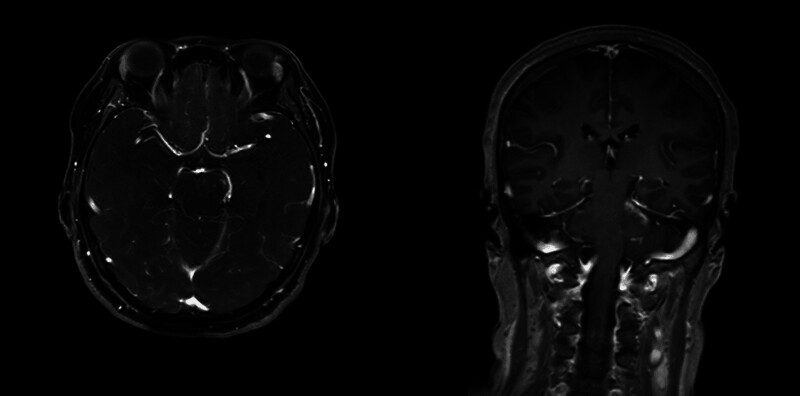
Brain MRI enhancement reveals abnormal enhancement foci in the bilateral pontine brachium, pons and soft meninges.

A previously healthy 52-year-old woman presented with diplopia and dizziness, and was admitted to our hospital 4 days after onset. There were no abnormalities on neurological examination, except for a slight nystagmus when viewing to the right with both eyes. The brain MRI showed abnormal hyperintense signals with unclear borders in the bilateral pontine brachium, predominantly on the right side (Fig. 1). Abnormal enhancement foci were observed in the bilateral pontine brachium and pons on MRI enhancement (Fig. 2). No obvious abnormalities were found in MRI plain scanning of both eyes and orbits. Ophthalmic examinations including fundus photography, optical coherence tomography, and synoptography revealed no evident abnormalities.

Routine serum chemistry including C-reactive protein showed normal results. Antinuclear antibodies were detectable at a titer of 1:80, but antibodies to double-stranded DNA, extractable nuclear antigens, onconeuronal antigens (Hu, Yo, Ri, CV2/CRMP5, Ma2/Ta, amphiphysin), cardiolipin, β2-glycoprotein, and phosphatidyl serine were negative. Cerebrospinal fluid examination showed elevation of Micrtotal protein (506 mg/L, 150–450 mg/L) and pleocytosis (8/μL, 87% lymphocytes). Oligoclonal IgG bands were negative. The IgG index was normal, and serum aquaporin-4 antibody was negative, while serum MOG-Ab was positive (1:100).

**Figure 3.**
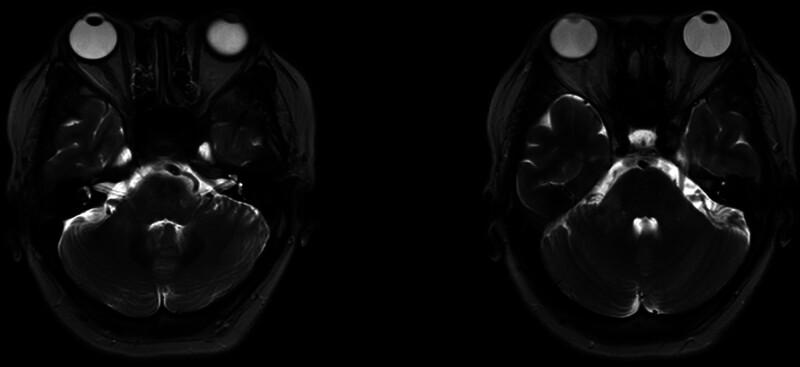
The abnormal signal in the left pontine brachium has resolved, and diminished in the right pontine brachium on T2-weighted fluid attenuated inversion recovery (T2-FLAIR) at the 4-month follow-up.

The patient was a middle-aged woman, with acute onset of the disease, mainly with double diplopia and dizziness, MRI: abnormal signals in bilateral pontine brachium, and serum MOG antibody positivity detected by cell-based assay with full-length human MOG as target antigen, while other diseases were excluded, and the final diagnosis of MOGAD was made. Subsequently, the patient was treated with corticosteroids: methylprednisolone sodium succinate was administered intravenously at a dose of 1000 mg/day for 3 days, after which the patient’s diplopia resolved. The treatment was then gradually tapered to oral prednisolone tablets at 10 mg/day, and maintained for 3 months, the titer of the serum MOG antibody was 1:10. Follow-up after 4 months showed disappearance of the abnormal signal on the left pontine brachium and diminished in the right side on T2-weighted fluid attenuated inversion recovery (Fig. 3), without any recurrence observed thus far 1 year.

## 3. Discussion

The pontine brachium, also known as the middle cerebellar peduncle, is the main afferent pathway of the cerebellum playing a crucial role in motor coordination. The etiology of pontine brachium lesions is complex and varied, involving cerebrovascular disease, demyelinating disease, neurodegenerative disease, tumors, infection, poisoning, and metabolic abnormalities.^[1,2]^ Due to different etiological factors, the severity of clinical manifestations varies. Our patient presented with acute dizziness and MRI found abnormal signals in bilateral pontine brachium, no previous risk factors for cerebrovascular disease or history of toxic substance exposure. Upon admission to the hospital, inflammatory indexes were within normal range. Brain MRI did not show any peripheral edema band or diffusion restriction on DWI. After excluding all possible differential diagnoses, the diagnosis of MOGAD was confirmed. Diplopia and dizziness are caused by lesions of the brainstem and pontine brachium. Brain enhancement MRI reveals foci of abnormal enhancement of the soft meninges. Although the meninges itself does not necessarily involve the peripheral nerves directly, it may be due to inflammation spreading to the oculomotor nerves, leading to the development of symptoms such as diplopia.

MOG is a protein exclusively expressed in the central nervous system on the surface of oligodendrocytes and their myelin sheaths.^[3]^ MOG has a role in regulating intercellular adhesion, skeletal proteins, and mediating myelin–immune cell associations.^[4]^ Recent advances in cell-based assay technology have led to the continued discovery and better understanding of MOG antibodies in idiopathic inflammatory demyelinating diseases.

MOGAD is a distinct disease entity from MS and NMOSD, with an expanding phenotypic spectrum that encompasses optic neuritis, transverse myelitis, encephalitis, seizures, and aseptic meningitis, adult prevalence is only 0.13/100,000.^[3]^ Brainstem or cerebellar involvement is present in approximately 34% of patients with MOGAD, with simple brainstem involvement being much rarer at only 5%.^[5]^ Lesions in patients with NMOSD are typically found in areas of high AQP4 expression, such as the hypothalamus, thalamus, or periventricular ependyma of the third ventricle, while periventricular and paraventricular lesions are more prevalent in MS than in MOGAD.^[6]^ Cobo-Calvo et al^[5]^ observed that the imaging presentation of MOGAD was dominated by thalamic and pontine lesions that were multifocal, poorly demarcated, and bilaterally involved. However, no specific imaging features were found in MOGAD compared to NMOSD.

Diffuse pontine and/or periventricular lesions adjacent to the fourth ventricle are more frequently in MOGAD, with a wide variation in clinical presentation, which aligns with our findings.^[7]^ A previous case report describing MOG-Ab-positive patients with MRI lesions similar to our cases.^[8]^ This patient initially presented with fever and impaired consciousness, and repeated MRI showed extensive brainstem edema with significant involvement of the bilateral pontine brachium and pons. Antiviral therapy was given on suspicion of acute meningitis, but nystagmus and quadriplegia with brain stem encephalitis rapidly developed over the next week. The diagnosis of MOGAD was finally confirmed by detection of positive MOG-specific antibodies in both serum and cerebrospinal fluid. Intravenous corticosteroids and immunoglobulin were then given immediately. The MRI changes disappeared and clinical symptoms regression dramatically during follow-up. The importance and urgency of early diagnosis and treatment is further emphasized by the rapid deterioration of the condition due to misdiagnosis of MOGAD patients in the study by Hang Shu.^[9]^

Patients with MOGAD are sensitive to glucocorticoids. Adequate doses of hormones administered in the acute phase often result in rapid and complete symptomatic reliefs, and early treatment is also associated with a favorable prognosis.^[3]^ A higher risk of relapse is associated with higher antibody titers and persistent serum antibody positivity over time.^[10]^ Olbert et al’s case report highlights the potential for relapse and disease progression in MOGAD patients.^[8]^ In this case, glucocorticoids were administered early in the course of the disease to patient who had mild clinical symptoms and a low serum MOG antibody titer. This effectively delayed the progression of the disease.

Currently, treatment of MOGAD includes symptom control in the acute phase and relapse prevention in the remission phase. Glucocorticoid therapy is preferred in the acute phase, and other treatments include intravenous immunoglobulin and plasma exchange.^[3]^ Prompt diagnosis at an early stage of the disease and treatment with massive glucocorticoid shock therapy had a rapid and complete resolution of the patient’s symptoms. Research has shown that about 2/3 of adults will suffer a recurrence.^[11]^ Maintaining low-dose hormones during remission may have reduced the risk of relapse. A higher risk of relapse is associated with higher antibody titers and persistent serum antibody positivity over time.^[10]^ In this case, glucocorticoids were administered early in the course of the disease to patient who had mild clinical symptoms and a low serum MOG antibody titer. This effectively delayed the progression of the disease.

Pontine brain lesions-abnormal signals in the bilateral pontine brachium have a complex etiology, and MOG antibody encephalitis has gradually been recognized as one of the causes, but the diagnosis is easy to miss. Although this patient is a classic case of MOG antibody encephalitis with brainstem lesions, the clinical manifestations of MOG antibody encephalitis are very heterogeneous, which needs to be observed and verified by a larger sample of cases and long-term follow-up. All in all, MOG antibody examination of a simple bilateral pontine encephalopathy with mild symptoms may provide important clues for the etiological diagnosis.

## 4. Conclusion

If brain MRI showing bilateral, multiple, and diffuse abnormal signals in the pontine brachium, and a discrepancy between the clinical symptoms and the imaging severity, a diagnosis of demyelinating disease should be considered highly probable. Anti-MOG antibody testing is essential for further defining the etiology. The clinical phenotype and imaging manifestations of MOG antibody-positive brainstem encephalitis may lack sufficient specificity to be readily identifiable. Timely diagnosis and early glucocorticoid therapy are beneficial in improving prognosis and preventing recurrence.

## Author contributions

**Conceptualization:** Fan-Ya Sun, Xiao-Tao Jia.

**Investigation:** Jia Shen, Le Ding, Bo Zhu, Naibing Gu, Zhiqin Liu.

**Writing – original draft:** Fan-Ya Sun.

**Writing – review & editing:** Fan-Ya Sun, Zhengli Di, Xiao-Tao Jia.
